# The Influence of Strychnine on the Urinary Organs

**Published:** 1847-07

**Authors:** 


*The Influence of Strychnine on the Urinary Organs.*—In several cases of paralysis affecting the lower extremities and the bladder, strychnine has been employed; and it has been remarked that it, in the first place, increases the urinary secretion, then causes very frequent desire to empty the bladder, and when this is done, it is attended with some smarting. This influence on the bladder declines in proportion as the effects of the strychnine manifest themselves in the muscles of the limbs.

In one case in which strychnine was given, a varioloid eruption came out, which did not suppurate, but terminated by crusty desiccations. When this eruption came out, the paralysis declined, and the bladder acquired power.

Strychnine, from the observations just mentioned, would therefore appear to exert a stimulant effect on the muscular tunic of the bladder; and if so, its utility would be rendered probable in paralytic conditions of the bladder, whether they be idiopathic, or arise from a mechanical cause ; and it would act as an adjuvant to other remedies, where a palsied state of the bladder is only symptomatic of other disease.—*Ibid.*

